# Enhanced 2,4-D Metabolism in Two Resistant *Papaver rhoeas* Populations from Spain

**DOI:** 10.3389/fpls.2017.01584

**Published:** 2017-09-13

**Authors:** Joel Torra, Antonia M. Rojano-Delgado, Jordi Rey-Caballero, Aritz Royo-Esnal, Maria L. Salas, Rafael De Prado

**Affiliations:** ^1^Department d'Hortofructicultura, Botànica i Jardineria, Agrotecnio, Universitat de Lleida Lleida, Spain; ^2^Department of Agricultural Chemistry and Edaphology, University of Córdoba Córdoba, Spain; ^3^DuPont de Nemours Paris, France

**Keywords:** degradation, malathion, plant detoxification process, non-target site resistance, sugar conjugate, synthetic auxin

## Abstract

Corn poppy (*Papaver rhoeas*), the most problematic broadleaf weed in winter cereals in Southern Europe, has developed resistance to the widely-used herbicide, 2,4-D. The first reported resistance mechanism in this species to 2,4-D was reduced translocation from treated leaves to the rest of the plant. However, the presence of other non-target site resistance (NTSR) mechanisms has not been investigated up to date. Therefore, the main objective of this research was to reveal if enhanced 2,4-D metabolism is also present in two Spanish resistant (R) populations to synthetic auxins. With this aim, HPLC experiments at two 2,4-D rates (600 and 2,400 g ai ha^−1^) were conducted to identify and quantify the metabolites produced and evaluate possible differences in 2,4-D degradation between resistant (R) and susceptible (S) plants. Secondarily, to determine the role of cytochrome P450 in the resistance response, dose-response experiments were performed using malathion as its inhibitor. Three populations were used: S, only 2,4-D R (R-703) and multiple R to 2,4-D and ALS inhibitors (R-213). HPLC studies indicated the presence of two hydroxy metabolites in these R populations in shoots and roots, which were not detected in S plants, at both rates. Therefore, enhanced metabolism becomes a new NTSR mechanism in these two *P. rhoeas* populations from Spain. Results from the dose-response experiments also showed that pre-treatment of R plants with the cytochrome P450 (P450) inhibitor malathion reversed the phenotype to 2,4-D from resistant to susceptible in both R populations. Therefore, it could be hypothesized that a malathion inhibited P450 is responsible of the formation of the hydroxy metabolites detected in the metabolism studies. This and previous research indicate that two resistant mechanisms to 2,4-D could be present in populations R-703 and R-213: reduced translocation and enhanced metabolism. Future experiments are required to confirm these hypotheses, understand the role of P450, and the relationship between both NTSR mechanisms. On this basis, selection pressure with synthetic auxins bears the risk of promoting the evolution enhanced metabolism in *Papaver rhoeas*.

## Introduction

Synthetic auxins were the first herbicidal mode of action discovered, back into 1940 (Peterson et al., 2016). 2,4-Dichlorophenoxyacetic acid (2,4-D) was the first herbicide belonging to this group to be commercially developed and released worldwide in 1945 (Schulz and Segobye, 2016). 2,4-D provided very effective control to the majority of broadleaved weed species in cereals, revolutionizing crop protection, and for this reason it was rapidly adopted by farmers in all developed countries (Peterson, 1967). In 1957, the first resistance cases were reported in North-America for *Daucus carota* and *Commelina diffusa* (Heap, 2017). Nowadays, after more than 70 years, 31 weed species are reported to have developed resistance to synthetic auxins, excluding monocotyledonous weeds (three species) resistant to quinclorac (quinoline-carboxylic acids). In total, there are 51 different reported cases with resistance to synthetic auxins worldwide. Of those, there are 31 reported cases with resistance to fenoxy-carboxylic acids (16 to 2,4-D), seven cases to benzoic acids (dicamba), and 13 different cases to pyridine-carboxylic acids (i.e., clopiralid; Heap, 2017). The rarity in occurrence of auxinic herbicide resistance compared to the hundreds of weed species that have evolved resistance to other herbicide classes, such as PS II- or ALS-inhibiting herbicides (Heap, 2017), could be attributed to: proposed multiple sites of action of these compounds (Mithila et al., 2011), initial low frequencies of resistant alleles, low levels of resistance conferred by resistance mechanism(s), or reduction in plant fitness due to pleiotropic effects of auxinic herbicide resistant traits (Busi and Powles, 2017). Single dominant nuclear encoded genes are supposed to control auxinic resistance in different species (Riar et al., 2011; Busi and Powles, 2017). However, polygenic inheritance of resistance in some species (Weinberg et al., 2006), could also contribute to slow evolutionary rates of auxinic herbicide resistance.

Plant detoxification processes usually follow a four-phase schema, which can also affect herbicides (Yuan et al., 2007). In phase I, molecules are activated for phase II enzymes. Oxidation is a typical phase I reaction, which can be carried out by cytochrome P450 monooxygenases. Phase II reactions generally involve conjugation (i.e., with sugars) which enables the end product to be recognized by the phase III transporters (usually ABC family), moving the molecule into the vacuole or extracellular space by active transport (Klein et al., 2006). Previous researches have proposed that the selectivity of auxinic herbicides in monocots is because of either limited translocation and/or rapid degradation of exogenous auxin, altered vascular anatomy, or altered perception of auxin (Peterson et al., 2016). It seems that the primary metabolic pathway in grasses is ester hydrolysis followed by the formation of base-labile 2,4-D conjugates (Hamburg et al., 2001). On the contrary, dicotyledonous species further detoxify auxinic herbicides in a different metabolic route after ester hydrolysis, mainly by means of ring hydroxylation, as it was observed in potatoes by Hamburg et al. (2001), mediated by cytochrome P450 (Hatzios et al., 2005).

Resistance mechanisms to synthetic auxins in weeds and their molecular basis remain largely unknown for most species. The main reason is that the precise mode of action of synthetic auxins is not fully understood (Grossmann, 2010). Moreover, some studies point out that these herbicides would have more than one target protein (multi-target; Mithila et al., 2011), partially explaining the polygenic characteristic of the resistant traits (Busi and Powles, 2017). Nonetheless, new discoveries including nuclear auxin receptors (F-box proteins), influx (AUX/LAX family) and efflux carriers (ABC and PIN families) and plasma membrane bound receptors (ABP proteins) have provided basic clues as to the molecular mode of action of these herbicides (Song, 2014).

In view of the complicated mode of action of auxinic herbicides, the evolution of resistance in weeds is generally treated as a non-target-site-based phenomenon (Goggin et al., 2016). Only one study considered a possible Target-site resistant (TSR) mechanism in *Brassica kaber*, due to an altered binding of auxinic herbicides to an auxin-binding protein (ABP) receptor located in plasma membrane (Mithila and Hall, 2005). Most studies indicate that Non-Target-site resistant (NTSR) mechanisms are involved in the majority of weed species. The lack of TSR mechanisms for this mode of action is explained by the central role that synthetic auxins targets (nuclear and membrane receptors or influx and efflux carriers) play in the gene expression, physiology and development of plants (Grossmann, 2010). Among the NTSR mechanisms, different absorption, translocation patterns, or herbicide metabolism between susceptible plants and resistant plants have been described in the few studied species (Peterson et al., 2016). Reduced absorption has been reported only in *Glechoma hederacea* (Kohler et al., 2004); reduced translocation has been reported in *Galeopsis tetrahit* (Weinberg et al., 2006), *Centaurea solstitialis* (Fuerst et al., 1996), *Lactuca serriola* (Riar et al., 2011), and in *Raphanus raphanistrum*, involving ABCB transporters in this later species (Goggin et al., 2016); increased translocation to the roots only in a *R. raphanistrum* biotype (Jugulam et al., 2013); while enhanced metabolism in *G. tetrahit* (Weinberg et al., 2006) and *Stellaria media* (Coupland et al., 1990). For example, mecoprop degradation could be mediated by a cytochrome P450 in *S. media* (Coupland et al., 1990).

*Papaver rhoeas* L. is the only known species to have evolved resistance to synthetic auxins in Spain. Though it was already reported in the early 90s (Taberner et al., 1995), their resistance mechanisms have only been studied very recently (Rey-Caballero et al., 2016). This research suggests that reduced 2,4-D translocation is involved in the resistance mechanism to synthetic auxins, likely leading to less ethylene production and greater survival in R plants. However, the presence of other NTSR mechanisms cannot be excluded, such as enhanced herbicide metabolism, because one resistant mechanism does not exclude the presence of others (Yu and Powles, 2014). Therefore, NTSR mechanisms to synthetic auxins, particularly enhanced metabolism, should be also investigated in *P. rhoeas* because, if presenttheir implication for integrated weed management can be tremendous (Yu and Powles, 2014). Enhanced detoxification pose a great threat to agriculture because of the often unexpected multi-herbicide resistance and multi-gene involvement in the mechanisms (Yuan et al., 2007).

The main aim of this research was to study if herbicide detoxification is also present in two 2,4-D resistant *P. rhoeas* populations: one only resistant to 2,4-D and the second multiple resistant to 2,4-D and tribenuron-methyl (Rey-Caballero et al., 2016). To do so, a new methodology using HPLC was developed, with the advantage that no radio labeled herbicide is required. Afterwards, two types of experiments were carried out: (1) HPLC experiments to find out differences in 2,4-D degradation between resistant and susceptible plants and identify and quantify the metabolites produced, and (2) dose-response experiments with a detoxifying enzyme (cytochrome P450) inhibitor (malathion) to further validate its possible role in 2,4-D degradation.

## Materials and methods

### Plant material

One susceptible (S) population (S-013) was included in this study, obtained from a seed dealer (Herbiseed, Twyford, UK) in 2008. The original field-evolved 2,4-D-resistant populations were collected from Almacelles (41°43′N, 0°27′E) in 2003 (population R-703) and Baldomar (41°54′N, 1°00′E) in 2013 (population R-213), both in North-eastern Spain; these populations displayed survival of ~20% (Rey-Caballero et al., 2016), respectively, when sprayed with the recommended field rate (600 g active ingredient/ha) of formulated 2,4-D ester. Additionally, R-213 was also resistant to ALS inhibiting herbicides (Rey-Caballero et al., 2017). Seeds were sown in aluminum trays with peat and placed in a growth chamber at 20/10°C day/night, 16 h photoperiod under 350 μmol photosynthetic photon-flux density m^−2^ s^−1^. After 14 days, seedlings were transplanted in 7 × 7 × 7 cm plastic pots filled with the following soil mixture: silty loam soil 40% (w/v), sand 30% (w/v), peat 30% (w/v). Pots were placed in a greenhouse in Lleida, north-eastern Spain (41°37′N, 0°38′E) and were watered regularly and fertilized as required.

### 2,4-D metabolism experiments

Seedlings from S and both R populations at six true leaves of development (5–6 cm) were treated at three different 2,4-D doses, 0, 600 g a.i.·ha^−1^ (field recommended rate, 1x) and 2,400 g a.i.·ha^−1^ (4x), as described below for the dose-response experiments. Six plants from each population and dose were harvested at 12, 24, 48, 96, and 168 h after treatment (HAT). Plants were separated into two parts: aerial part (leaves and shoots) and roots, each of which was rinsed using distilled water to remove unabsorbed herbicide. Each part was rapidly frozen in liquid nitrogen and then stored at −40°C until use.

To study the 2,4-D metabolism in *P. rhoeas*, some known methodologies were used (Chkanikov et al., 1977; Hamburg et al., 2001) to confirm the existence or not of its metabolites in the populations. These methodologies were adapted and modified to be able to work without radiolabelled herbicide, because it was not possible to obtain ^14^C-2,4-D metabolites. The inability to obtain the ^14^C-2,4-D-metabolites required identification and quantification by a chromatographic method. This method was based on that one by Hamburg et al. (2001), which also was used to identify the non-radiolabelled metabolites, according to the retention times. All details regarding how these methodologies were adapted and modified are provided below.

#### Reagents

Acetone (HPLC grade), acetic acid, chlorhydric acid (37%), 1-butanol, diethyl ether, ethanol (HPLC grade), petroleum ether, and 2,4-D herbicide standard were purchased from Sigma Aldrich (St Louis, MO, EE.UU.); acetonitrile, methanol (LC-MS grade) and ammonium hydroxide from Panreac AppliChem (Barcelona, Spain). Deionised water (18 MΩ·cm) was obtained with a purification system Millipore Milli-Q (Millipore, Bedford, MA, EE.UU.). Commercial 2,4-D herbicide was Esteron 60 (60% w/v, Dow AgroSciences).

#### Instruments and apparatus

A magnetic stirrer with a temperature controller from Selecta (Barcelona, Spain) was used for some separation phase steps. Centrifugation of the extracts was carried out by a Coulter Avanti J-25 centrifuge with a temperature controller (Beckman, Fullerton, USA). A rotatory evaporator Mod. LABOROTA 4000 from Heidolph (Schwabach, Germany) was used for the organic solvent evaporation. The 20 × 20 cm silica gel TLC plates with inorganic fluorescent indicator F254 from Merck Millipore (Billerica, MA, USA) was used for separation steps. Nylon filters with a pore size of 20 μm and an inner diameter of 13 mm from Millipore (Billerica, MA, USA) were used to remove solid particles from the extracts before the LC analysis. A 15 Gold LC System from Beckman Coulter (Fullerton, USA) equipped with a 26 Gold DAD detector (wavelength range 190–600 nm) was used for individual separation and UV detection. The instrumental setup was controlled by the Karat 3.0.7 software, which also enabled data acquisition and processing. Chromatographic separation was carried out using a Kinetex® EVO C18 column (150 mm, 4.6 mm id, 2.6 μm particle size) from Phenomenex Inc. (Torrance, CA, USA), furnished with a 4.6 mm SecurityGuard™ ULTRA cartridges.

#### Sample pre-treatment and first partition

The extraction followed the methodology described by Chkanikov et al. (1977) with some modifications for the full extraction. Frozen samples were washed with 5 mL of 0.05 N of ammonium hydroxide. Each sample was placed in a porcelain mortar and flash-frozen using 20 mL liquid nitrogen and grinded to fine homogeneous powder using a porcelain pestle for 5 min. Then it was submerged in boiling water (three times) and the aqueous extracts were combined, cooled, and an equal volume of acetone was added. After 12 h the formed precipitate was removed using centrifugation at 4°C and 20,000 rpm. The precipitate was washing three times with 5 mL of ethanol and added to the acetone phase. The organic phase (ethanol and acetone) was removed at 40°C with a rotary evaporator. The aqueous residue was acidified to pH 2 with hydrochloric acid. It was treated three times with 5 mL of diethyl ether and evaporated this ether portion in the rotary evaporator at 40°C.

After the ether portion was evaporated, the residue resulting was dissolved in 90% acetone and an aliquot of this was developed by TLC in the solvent butanol-ammonium hydroxide-water (5:1:4) (First partition). Unaltered 2,4-D (Rf 0.55) was separated from its “free” hydroxylated derivatives (Rf 0.2) and amino acid conjugates (Rf 0.1).

#### Second partition

A second partition was realized using the acidified aqueous phase with1-butanol for the extraction, after the diethyl ether phase was removed. The 1-butanol was later evaporated in the rotary unit at 40°C. The residue was dissolved in 2 N hydrochloric acid and then hydrolyzed for 60 min in a boiling water bath. The metabolites were separated by TLC in the same solvents. As a result of hydrolysis l-O-(2,4-dichlorophenoxyacetyl)-ß-D-glucose broke down with the release of unaltered 2,4-D, while 4-O-ß-D-glucosides of 4-hydroxy-2,5-dichloro- and 4-hydroxy-2,3-dichlorophenoxyacetic acids released “free” 4-OH-2,5-D and 4-OH-2,3-D.

#### Third partition

The third partition was realized using the extract with the 1-butanol totally evaporated, and hydrolyzed in 2 N hydrochloric acid at 100°C. The distillate was acidified with hydrochloric acid to pH 1 and was extracted with petroleum ether (40°C) in the rotatory unit. The substances were separated by TLC with the same solvents. A glycoside of 2,4-dichlorophenol was detected in the analysis of ring-labeled-herbicide-treated strawberry plants known for an extremely high rate of 2,4-D ether linkage breakdown. After TLC development, the TLC plates were exposed to a UV lamp at 256 nm for discover the metabolites and could be separated. The metabolites were scratched from the TLC plates (dark areas) and dissolved in 0.5 mL of acetone. The reconstituted sample was filtered through a nylon filter syringe before chromatographic analysis.

#### Chromatographic method

The chromatographic method was the method used by Hamburg et al. (2001) with some modifications, which also was used to identify the metabolites according to the retention times. Fifty microliters of the reconstituted simple was injected in the HPLC system. 1% (v/v) acetic acid in water and acetonitrile as mobile phases A and B, respectively, were used. The elution program started by a linear gradient from 20% mobile phase B to 50% in 20 min (step 1), 50% mobile phase B to 100% in 5 min (step 2), and 100 to 10% acetonitrile in 10 min for equilibration (step 3). The constant flow rate and column temperature were 1.0 mL/min and 25°C, respectively. Quantification of 2,4-D and its metabolites was based on the calibration curve of 2,4-D, which is the unique commercially available standard. The results were expressed as μg of the analyte/g of plant.

### Dose-response experiments

Five seedlings were sown per pot and after establishing, were thinned to four per pot. At the four to six leaf stage (4–5 cm), all populations were treated with either 0 or 2,000 g a.i./ha of the organophosphate insecticide malathion ([(dimethoxyphosphinothioyl)-thio] butanedioic acid diethyl ester). Preliminary tests showed that 2,000 g a.i./ha is around the maximum dose not affecting *P. rhoeas* survival or growth (data not shown). After approximately 1 h 30 min, 2,4-D (Esteron 60, Dow AgroSciences, 60%) was applied at 0, 300, 450, 600 (field dose), 900, 2,400, and 4,800 g a.i./ha to R populations and at 0, 225, 300, 450, 600, 900, and 2,400 g a.i./ha to S plants. Non-treated plants were used as controls. A total of four replicates (four plants per pot) were included at each dose. Herbicides were applied using a precision bench sprayer delivering 200 L/ha, at a pressure of 215 kPa. Four weeks after treatment, percentage of survival was estimated, and plants were harvested (above ground) and the dry weight (65°C for 48 h) was measured. The experiment was repeated twice.

### Statistical analysis

Data from dose-response experiments were analyzed using a nonlinear regression model (1). The herbicide rate required for 50% growth reduction of plants (GR50) was calculated with the use of a four parameter logistic curve of the type:

$$y=c+\frac{\left(d-c\right)}{1+\text{EXP}[b(\log\left(x\right)-\log\left(XR50\right)]}$$

where *c* = the lower limit set to 0, *d* = the upper limit set to 100, and *b* = the slope at the XR50 (SR50 for % of survival and GR50 for % of dry weight compared to untreated control). In this regression equation, the herbicide rate (g a.i./ha) was the independent variable (x) and the plants survival and the plants' dry weight expressed as percentage of the untreated control were the dependent variables (y). The resistance index (RI) was computed as GR50(R)/GR50(S). XR50 parameters were compared between susceptible and R populations (with and without malathion) with the Delta method at *P* = 0.05. Repetitions from the dose-response experiments were pooled due to lack of statistical differences between them. Data from 2,4-D metabolism experiment was subjected to analysis of variance (ANOVA). The requirement of homogeneity of variance was checked by visual inspection of the residual plots and residuals were analyzed using Shapiro–Wilk Test. When required, data were previously squared root transformed; in those few cases non-transformed values are presented for clarity. Where variances were not homogeneous, generalized linear models (GLM) were used. The binomial distribution (Logit-link) was used in all GLM, because this distribution resulted in normally distributed residues. Population means were compared using a *post-hoc* Tukey's pairwise procedure at *P* = 0.05.

All statistical analyses were carried out with the use of the R programming language (R Core Team, 2013), drc package (Knezevic et al., 2007) for the non-linear regression and multcom (Hothorn et al., 2008) for the *post-hoc* Tukey's test were employed.

## Results

### 2,4-D metabolism experiments

Qualitative assessment of TLC bands showed differences in the migration patterns between the studied populations (Supplementary Figure 1). In the S population (Supplementary Figure 1A), only parent 2,4-D migrating identically to the standard was detected at all times from 0 to 168 HAT. On the other hand, from 24 HAT, migration patterns were different in both R populations. In the 2,4-D resistant population (Supplementary Figure 1B), compounds remaining close to the origin were already detected at 24 HAT in plants applied at 2,400 g a.i./ha (4x), while in the multiple resistant population (Supplementary Figure 1C) they were detected at 48 HAT at both doses. Interestingly, another compound appeared in the multiple resistant population at 96 and 168 HAT, even closer to the origin.

Quantification of the relative abundance of TLC bands of parent 2,4-D and its metabolites showed clear differences in 2,4-D metabolic capacity between the S and R *P. rhoeas* populations; so dots on TLC plates were due to compounds migrating differentially (less) to parent 2,4-D (Table 1). Up to 48 HAT, amounts of 2,4-D in the aerial part were similar between S and R plants at both rates. At 96 HAT significantly much less 2,4-D was detected in R populations, while at 168 HAT no parent 2,4-D was found. At 12 HAT the herbicide was already found in roots in all populations, but quantities were much higher in S plants in all assessment times. Ascribed 2,4-D metabolites (according to HPLC retention times) were only quantified in the R populations. In the R-703 population (2,4-D resistant), the first metabolite (2,3-D) was detected in aerial parts already 24 HAT and 48 HAT, at 4x and 1x rates, respectively, while in the R-213 population (multiple R), it was detected at 48 and 96 HAT, respectively. In roots of R-703 population, it was already detected 24 and 96 HAT, at 4x and 1x rates, respectively, while in R-213 it was detected at 48 and 168 HAT, respectively. The second metabolite (2,5-D), was first detected in the aerial parts at 48 HAT in both R populations at 4x, and at 96 HAT at 1x. Interestingly, this compound was only detected in roots of the multiple R population at 96 and 168 HAT at the highest dose. Finally, a third compound (a sugar conjugate) was only quantifiable in the roots of the 2,4-D R population at 96 and 168 HAT at the highest rate, while in the aerial only in the last assessment time.

**Table 1:** Amount (μg/g plant) of 2,4-D and its metabolites of one susceptible (S) and two resistant (R-703 and R-213) *Papaver rhoeas* populations at 12, 24, 48, 96, and 168 HAT applied at two doses (1x for 600 g/ha; 4x for 2,400 g/ha).

**Dose**	**Part**	**Product**	**Pop**	**12**	**24**	**48**	**96**	**168**
1x	Aerial part	2,4-D	S	8.6a^*^ (0.1)	9.2a (0.0)	10.7a (0.0)	10.7a (0.2)	9.3 (0.1)
			R-703	7.6b (0.0)	8.2c (0.0)	9.2b (0.0)	5.0c (0.1)	ND
			R-213	7.7b (0.1)	9.0b (0.0)	11.0a (0.2)	7.0b (0.2)	ND
		2,3-D	S	ND	ND	ND	ND	ND
			R-703	ND	ND	2.4 (0.0)	5.1a (0.1)	7.6a (0.1)
			R-213	ND	ND	ND	2.7b (0.1)	7.7a (0.3)
		2,5-D	S	ND	ND	ND	ND	ND
			R-703	ND	ND	ND	1.61a (0.05)	3.59a (0.05)
			R-213	ND	ND	ND	0.86b (0.03)	3.64a (0.13)
		Sugar conjugate	S	ND	ND	ND	ND	ND
			R-703	ND	ND	ND	ND	ND
			R-213	ND	ND	ND	ND	ND
	Root	2,4-D	S	0.2a (0.0)	0.3a (0.0)	0.7a (0.0)	0.9a (0.0)	2.4 (0.0)
			R-703	0.2b (0.0)	0.1b (0.0)	0.1c (0.0)	0.0c (0.0)	ND
			R-213	0.1b (0.0)	0.1b (0.0)	0.1b (0.0)	0.1b (0.0)	ND
		2,3-D	S	ND	ND	ND	ND	ND
			R-703	ND	ND	ND	0.02 (0.0)	0.03b (0.0)
			R-213	ND	ND	ND	ND	0.05a (0.0)
		2,5-D	S	ND	ND	ND	ND	ND
			R-703	ND	ND	ND	ND	ND
			R-213	ND	ND	ND	ND	ND
		Sugar conjugate	S	ND	ND	ND	ND	ND
			R-703	ND	ND	ND	ND	ND
			R-213	ND	ND	ND	ND	ND
4x	Aerial part	2,4-D	S	32.0a (0.0)	33.2b (0.1)	38.4a (0.1)	38.9a (0.1)	33.3a (0.5)
			R-703	31.7a (0.1)	33.1b (0.2)	35.2b (0.1)	13.3c (0.1)	0.0b (0.0)
			R-213	31.8a (0.2)	35.9a (0.1)	29.0c (0.9)	15.5b (0.1)	0.1b (0.0)
		2,3-D	S	ND	ND	ND	ND	ND
			R-703	ND	1.4 (0.1)	6.7b (0.0)	27.4b (0.1)	35.7a (0.1)
			R-213	ND	ND	10.4a (0.3)	22.8a (0.2)	34.5b (0.2)
		2,5-D	S	ND	ND	ND	ND	ND
			R-703	ND	ND	3.28b (0.01)	7.29a (0.03)	10.07a (0.03)
			R-213	ND	ND	5.15a (0.16)	6.06b (0.05)	9.99a (0.07)
		Sugar conjugate	S	ND	ND	ND	ND	ND
			R-703	ND	ND	ND	ND	ND
			R-213	ND	ND	ND	ND	0.91 (0.0)
	Root	2,4-D	S	0.7a (0.0)	1.2a (0.0)	2.7a (0.0)	3.4a (0.0)	8.9 (0.4)
			R-703	0.1c (0.0)	0.3b (0.0)	0.3c (0.0)	0.0c (0.0)	ND
			R-213	0.2b (0.0)	0.3b (0.0)	0.3b (0.0)	0.2b (0.0)	ND
		2,3-D	S	ND	ND	ND	ND	ND
			R-703	ND	ND	0.04b (0.0)	0.1b (0.0)	0.16b (0.01)
			R-213	ND	ND	0.17a (0.0)	0.3a (0.0)	0.47a (0.03)
		2,5-D	S	ND	ND	ND	ND	ND
			R-703	ND	ND	ND	ND	ND
			R-213	ND	ND	ND	0.09 (0.0)	0.14 (0.01)
		Sugar conjugate	S	ND	ND	ND	ND	ND
			R-703	ND	ND	ND	ND	ND
			R-213	ND	ND	ND	ND	0.01 (0.0)

* *Means within a column, evaluation time, plant part and product followed by the same letter are not significantly different (P > 0.05)*.

**Figure 1:**
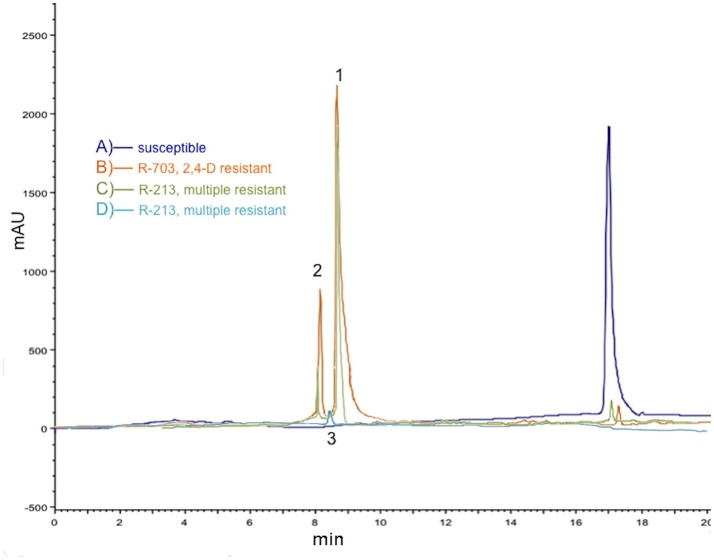
Comparison of 2,4-D metabolism in three *Papaver rhoeas* populations in 2,4-D treated plants (2,400 g ai./ha after 168 HAT). **(A)** Representative HPLC chromatogram of extract (second partition) from S plants in purple; only 2,4-D was detected. **(B)** Representative HPLC chromatogram of extract (second partition) from only 2,4-D R plants (population R-703) in orange; 2,4-D and two metabolites, 2,3-D (1) and 2,5-D (2) were detected. **(C)** Representative HPLC chromatogram of extract (second partition) from multiple R plants (population R-213) in green; 2,4-D and two metabolites, 2,3-D (1) and 2,5-D (2) were detected. **(D)** Representative HPLC chromatogram of extract (third partition into ether phase) from multiple R plants in blue; a sugar conjugated compound (3) was detected. Representative HPLC chromatograms from three independent experiments are shown.

Qualitative and quantitative differences were found between the populations in the HPLC profile of 2,4-D metabolism (Figures 1A–C). As expected, 2,4-D had a retention time on HPLC of ~17 min in both S and R populations. *P. rhoeas* R populations produced a mixture of two polar metabolites (which remained near the origin on TLC), with HPLC retention times of 8 and 9 min, respectively, which were water soluble (Figures 1B,C). Additionally, very small amounts of another polar metabolite (smallest dots even closer to origin in TLC plates) had an HPLC retention time around 8.5 min, which was partitioned into the ether phase (Figure 1D). The two hydroxilated metabolites (2,3-D and 2,5-D) were detected at 168 HAT in the two R populations (2,4-D and multiple resistant), while in the sugar conjugate was only detected in the multiple R population. The amount of the 2,4-D found in S plants was higher (>10-folds) than in R plants (Figure 1), while the metabolites were not detected in S plants. With respect to the amount of the different metabolites between the populations, the first polar compound (2,3-D) was detected in similar quantities in both R populations, while the levels of the second compound (2,5-D) were 2.5-folds higher in the 2,4-D R population compared to the multiple R one.

Summarizing, the 2,4-D was rapidly degraded, through the hydroxylation of the phenyl ring generating 4-hydroxy-2,5-dichlorophenoxyacetic acid (2,5-D) and 4-hydroxy-2,3-dicholorophenoxyacetic acid (2,3-D), which were not present in S plants but were in the two R populations in significant amounts. The third metabolite, a sugar conjugate, might be a conjugation of OH-2,5-D with a carbohydrate. Sugar conjugates did not appear in R-703 (only 2,4-D R) and S plants (Table 1). Both 2,3-D and 2,5-D metabolites and the sugar conjugate are regarded as being far less phytotoxic than 2,4-D (Peterson et al., 2016). The three metabolites were ascribed to those previously appointed according to retentions times in the HPLC, but further identification with other methodologies, i.e., mass spectrometry, would be required.

### Dose-response experiments

**Figure 2:**
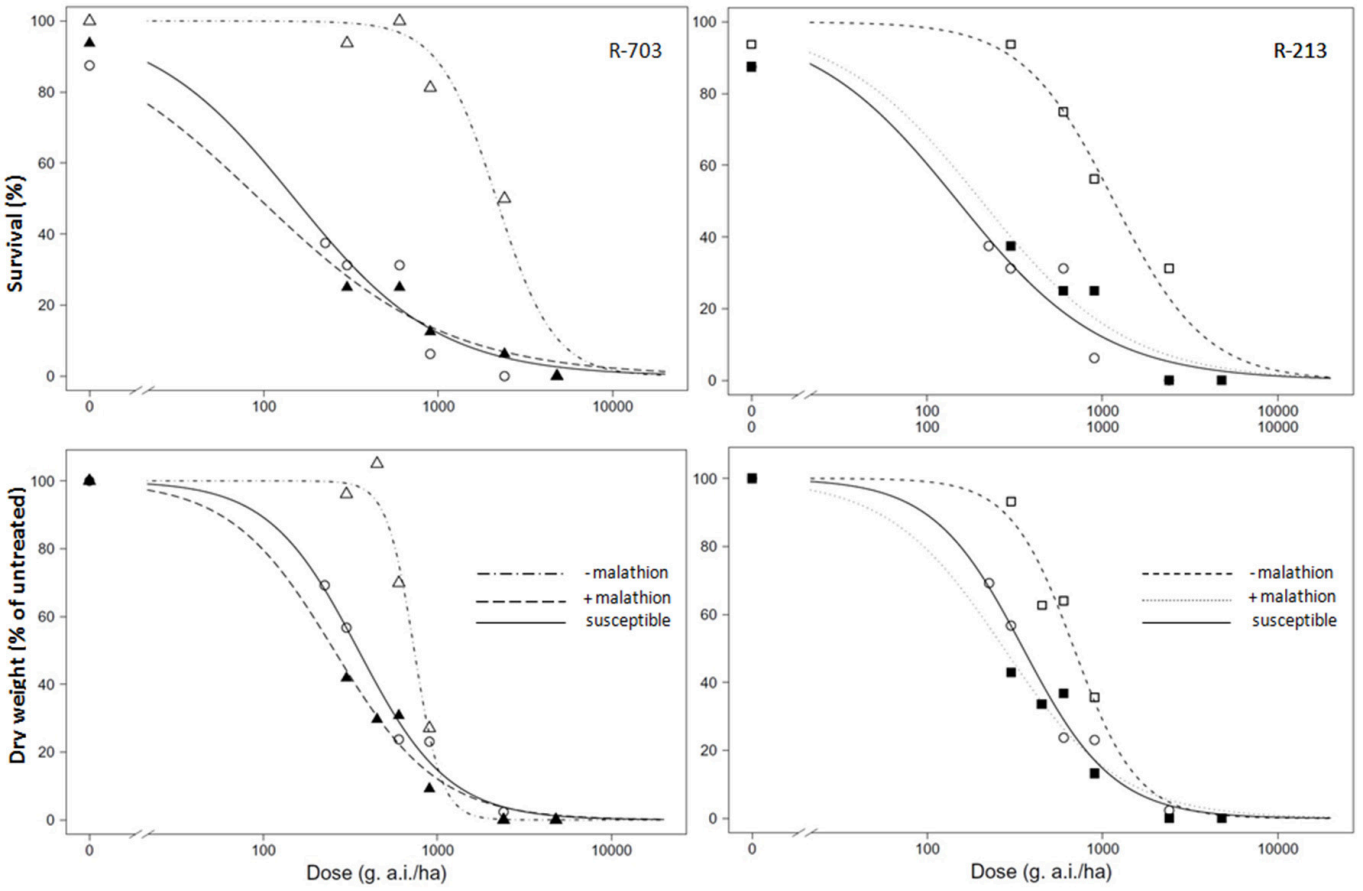
Dose-response regression curves of 2,4-D on log scale without malathion or with addition of malathion at the dose 2,000 g/ha (+malathion) in susceptible and resistant populations R-703 (**left** side, 2,4-D resistant) and R-213 (**right** side, multiple resistant) of *Papaver rhoeas*. Up line, percentage of survival; Bottom line, percentage of the mean dry weight of untreated control plants. Dashed (R populations) and solid (susceptible) lines represent predicted values derived from the regression analysis.

When malathion was applied alone at 2,000 g/ha, there was no effect on survival or growth of either the S or R populations. When 2,4-D was applied after malathion on the susceptible population, the behavior in terms of survival and biomass were similar without the presence of the insecticide (Table 2). In the presence of malathion, both R populations became susceptible to 2,4-D (Figure 2), and the RI for % of survival went done from 14 to 0.6 and from 8 to 1.4, for the 2,4-D resistant and the multiple resistant populations, respectively (Table 2). Similar results were obtained for the above-ground biomass (Figure 2 and Table 2).

**Table 2:** Equation parameters of the log-logistic models used to estimate dose-response regression curves (% Survival and % Dry weight of untreated control) in susceptible and auxin resistant populations (R-703 and R-213) for 2,4-D with (+ malathion) or without (− malathion) previous application of malathion at 2,000 g/ha.

**Parameter**	**Population**	**Treatment**	X50 ± SE (g a.i./ha)^a^^*^	**B ± SE^b^**	**Res SS^c^**	**RI^d^**
% Survival	Susceptible	− malathion	150 ± 53*a*	1.0 ± 0.4	242	1
		+ malathion	125 ± 45*a*	0.9 ± 0.3	253	0.8
	R-703	− malathion	2154 ± 283*b*	2.7 ± 0.8	13,906	14
		+ malathion	94 ± 84*a*	0.8 ± 0.4	52	0.6
	R-213	− malathion	1164 ± 170*b*	1.7 ± 0.3	1,463	8
		+ malathion	205 ± 85*a*	1.0 ± 0.4	190	1.4
Dry weight (% of untreated)	Susceptible	− malathion	352 ± 51*a*	1.7 ± 0.5	127	1
		+ malathion	301 ± 47*a*	1.6 ± 0.4	154	0.9
	R-703	− malathion	736 ± 52*b*	5.5 ± 1.5	5	2
		+ malathion	255 ± 74*a*	1.5 ± 0.6	156	0.7
	R-213	− malathion	687 ± 84*b*	2.4 ± 0.7	51	2
		+ malathion	274 ± 85*a*	1.3 ± 0.5	592	0.8

* *Means within a column, evaluation time, plant part and product followed by the same letter are not significantly different (P > 0.05)*.

a *XR50, herbicide concentration for 50% reduction of corn poppy survival and dry weight*.

b *Slope at the XR50*.

c *Res SS, residual sum of squares*.

d *RI (resistance index) = GR 50(Population) ÷ GR50(susceptible)*.

**Figure 3:**
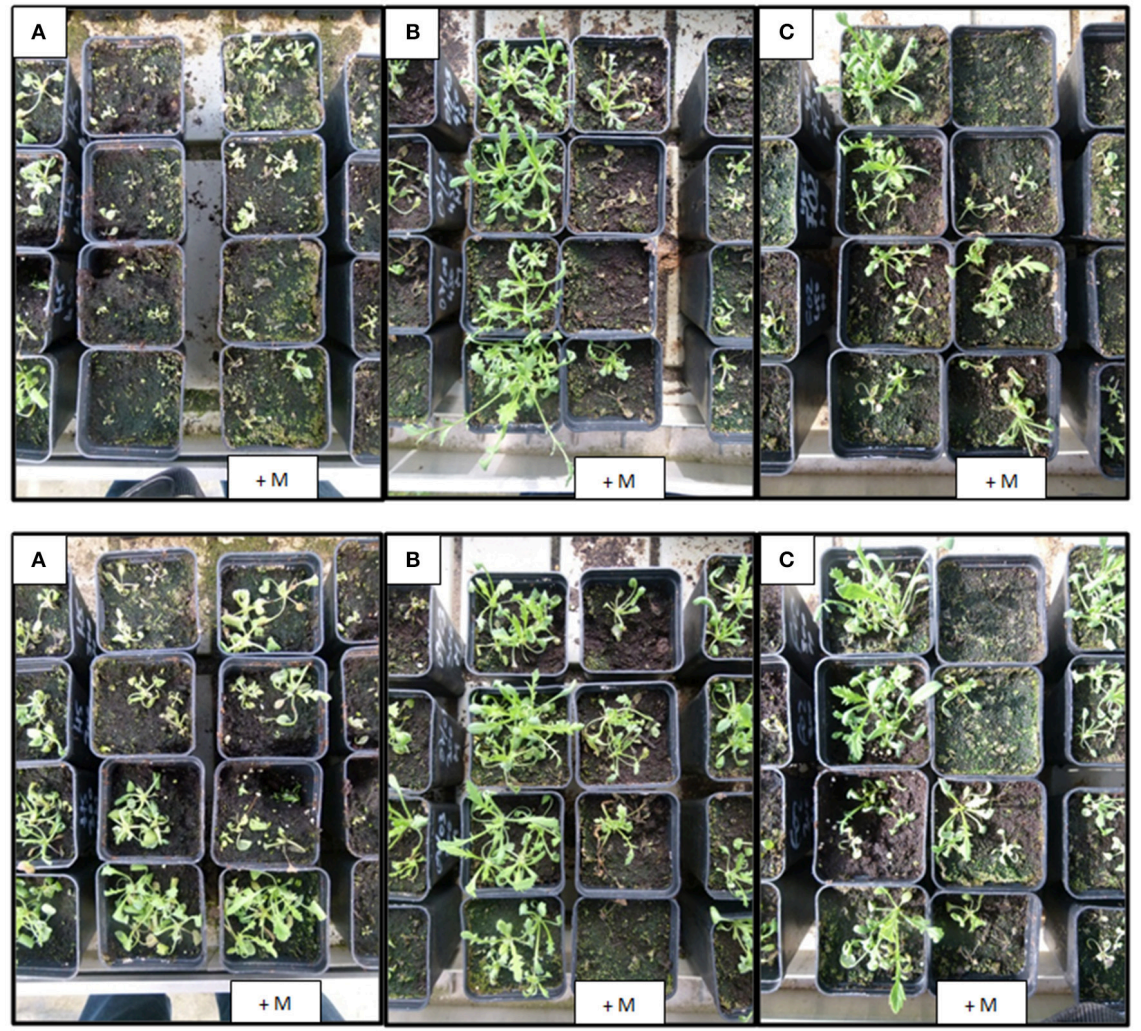
Visual injury of three *Papaver rhoeas* populations 14 days after treatment with 2,4-D and with (+ M) or without previous malathion application at 2,000 g a.i./ha. Up line: 600 g a.i./ha; Bottom line: 450 g a.i./ha. Left **(A)**, susceptible population; Middle **(B)**, only 2,4-D R population (R-703); Right **(C)**, multiple R population (R-213).

Visual inspection of treated plants of both R populations comparing both treatments, that is with or without previous application of malathion, clearly revealed that survival and growth were much reduced with previous applications of the insecticide (Figure 3).

## Discussion

In Spain, cereals (mainly rainfed winter barley and wheat) are most extended crops, with more than 6 million hectares in 2015. *P. rhoeas* is the most troublesome broadleaved weed in these crops due to the spread of multiple R populations to synthetic auxins and ALS inhibiting herbicides. This research is pioneer in studying the presence of enhanced 2,4-D metabolism in this species. For the first time, the presence of two 2,4-D hydroxy metabolites (2,3-D and 2,5-D) has been indicated in two R *P. rhoeas* populations, one only 2,4-D R and another one multiple R, while none were detected in S plants. Therefore, enhanced metabolism to synthetic auxins becomes a newly discovered resistance mechanism in this species. Other few reports of enhanced metabolism to phenoxyacetic acid herbicides included *Stellaria media, R. raphanistrum*, and *G. tetrahit* (Coupland et al., 1990; Weinberg et al., 2006; Goggin and Powles, 2014).

Results from the dose-response experiments showed that pre-treatment of R plants with the cytochrome P450 (P450) inhibitor malathion clearly reversed the phenotype to 2,4-D from resistant to susceptible in both R populations. These dramatically visual effects on survival and growth in R plants provided indirect evidence that differential activity of a P450 mono-oxygenase (inhibited by malathion) is required for the resistance response in *P. rhoeas*. Enhanced metabolism mediated by the cythochrome P450 family was postulated in *S. media* for mecoprop (Yuan et al., 2007). Therefore, it is hypothesized that the 2,4-D hydroxy metabolites detected in the metabolism experiments as result of enhanced metabolism could be due to the enhanced activity of a P450. However, this interpretation should be investigated and confirmed in the future. For example, a characterization of the possible P450 involved in the resistance response using different inhibitors (malathion, 1-aminobenzotriazole, piperonyl butoxide, or tetcyclasis) would be of value. Another issue to consider is whether this suspected enhanced metabolism in these two R *P. rhoeas* populations could detoxify or not herbicides from other modes of action, leading to cross-resistance or multiple resistance (Preston, 2004; Yu and Powles, 2014). In Spain, multiple resistant populations to ALS inhibitors and synthetic auxins were already reported back into the 90s (Rey-Caballero et al., 2017). But since then, cross-resistance cases to any other mode of action have not been reported in this species (Heap, 2017). Specificity of enzymes responsible of metabolic resistance to a given herbicide might explain the lack of cross resistances to other modes of action (Yu and Powles, 2014). A previous study reported ALS inhibitors enhanced metabolism in multiple R *P. rhoeas* from Spain (Rey-Caballero et al., 2017). Remains to be investigated if the detoxifying mechanisms in multiple R populations to 2,4-D and ALS inhibiting herbicides are linked or evolved independently.

Diverse NTSR mechanisms, including enhanced metabolism (Coupland et al., 1990) and decreased translocation (Jugulam et al., 2013; Goggin et al., 2016), have been reported in R weeds to auxinic herbicides. For example, *L. serriola* L. and *R. raphanistrum* resistant to 2,4-D displayed reduced uptake and translocation compared with S populations, but rates of 2,4-D metabolism were not different (Riar et al., 2011; Goggin et al., 2016). On the other hand, in MCPA-resistant *G. tetrahit* it was suggested that lower rate of MCPA translocation and a higher rate of MCPA metabolism in the roots were two different R mechanisms, as the inheritance of MCPA resistance was governed by at least two nuclear genes with additive effects (Weinberg et al., 2006). Interestingly, reduced translocation was also described in the same two *P. rhoeas* R populations used in this study (Rey-Caballero et al., 2016). So, it is hypothesized that two resistance mechanisms are present in these two populations, reduced translocation and herbicide degradation. How are they related and which is the primary mechanism remains unknown. One possibility is that 2,4-D metabolites could lead to decreased translocation due to less phloem mobility than parent compound (Han et al., 2013), or less likely due to permanent sequestration, i.e., in the vacuole via phase III ABC transporters (Riechers et al., 2010). More likely, it could be speculated that the impaired 2,4-D transport observed in previous studies (Rey-Caballero et al., 2016) is due to an alteration efflux ABCB transporters (auxin long-distance movement) preventing herbicide loading into phloem and its movement in resistant plants. The role of ABCB family in impaired transporter has been proposed for some species (Goggin et al., 2016; Kuepper et al., 2017). Afterwards, while 2,4-D accumulation is occurring within cells cytoplasm, enhanced herbicide metabolism might start, a degrading route involving ring hydroxylation by means of a P450 in phase I. Again, new experiments are required to validate these statements and understand the relationship between these two resistance mechanisms.

A variety of metabolic degradation pathways for 2,4-D are known in plants and include side-chain degradation, side-chain lengthening, ring hydroxylation, conjugation, and ring cleavage (Riar et al., 2011). The presence of a sugar conjugate in the multiple R population (not in the 2,4-D R one) currently remains speculative, but it could be construed as an additional reaction in the plant detoxification processes. It is likely that the ascribed 2,4-D hydroxy metabolites were phase I products, that is, ring or methyl hydroxylates, whereas the third metabolite found in shoots and roots of the multiple resistant population (only at 168 HAT) could be a result of a second phase II reaction, that is, sugar conjugation, as ascribed afterwards by HPLC. This reaction might involve a glucosyl transferase enzyme (GT), which catalyzes a glucose conjugation and has been postulated as an enzyme implied in the enhanced metabolism observed in other resistant cases (Yu and Powles, 2014). This hypothesis could not indirectly be confirmed in this research, since to best of our knowledge, there are not known GT inhibitors to be used in dose-response experiments with whole plants. The sugar conjugate appeared to be mobile within *P. rhoeas* plants, since it was found both in shoots and, to a lesser amount, in roots. Considering that very small amounts of the sugar conjugate were detected only in the last assessment time, its presence should not be discharged in the only 2,4-D R population after longer evaluations times. An interesting report involving MCPA-resistant *R. raphanistrum* demonstrated increased MCPA translocation to roots in the resistant population (in the absence of altered metabolism), which may have been related to extrusion of parent herbicide out of the roots into the soil (Jugulam et al., 2013). A root-localized ABC transporter (ABCB4) could play a role, as *Arabidopsis* abcb4 mutants were resistant to moderate concentrations of 2,4-D (Goggin et al., 2016).

In conclusion, this is the first study reporting enhanced 2,4-D metabolism in *P. rhoeas* in two R populations. According to the results presented in this research, we propose that the observed enhanced metabolism is mediated by a cytochrome P450. Resistance in this species is not only due to reduced translocation to target sites, as shown in a previous study with these same populations (Rey-Caballero et al., 2016), but also due to enhanced metabolism. So far, it is unknown which is the relative importance of each mechanism in the resistance response and how they are physiologically related. Future research, including inheritance studies and transcriptome analyses, should help elucidate the hypotheses stated in this research, the number of responsible genes and the potential risk of cross-resistance to other modes of action.

## Author contributions

MS: Secured the funding; JT, AR-D, JR, MS, and RD: Idea and designed the experiments; JT, AR-D, JR, and AR-E: Performed the research; JT, AR-D, JR, AR-E, and RD: Interpretation and analysis of results (of raw data); JT, AR-D, JR, AR-E, MS, and RD: Wrote and approved the manuscript.

### Conflict of interest statement

The authors declare that the research was conducted in the absence of any commercial or financial relationships that could be construed as a potential conflict of interest.
