# Supplementary material for: Enhanced 2,4-D Metabolism in Two Resistant Papaver rhoeas Populations from Spain
Source: Front Plant Sci. 2017 Sep 13;8:1584. doi: 10.3389/fpls.2017.01584 (PMC5602352; doi:10.3389/fpls.2017.01584)
Supplement: Supplementary file 1 [file Image1.PDF]

Supplementary material

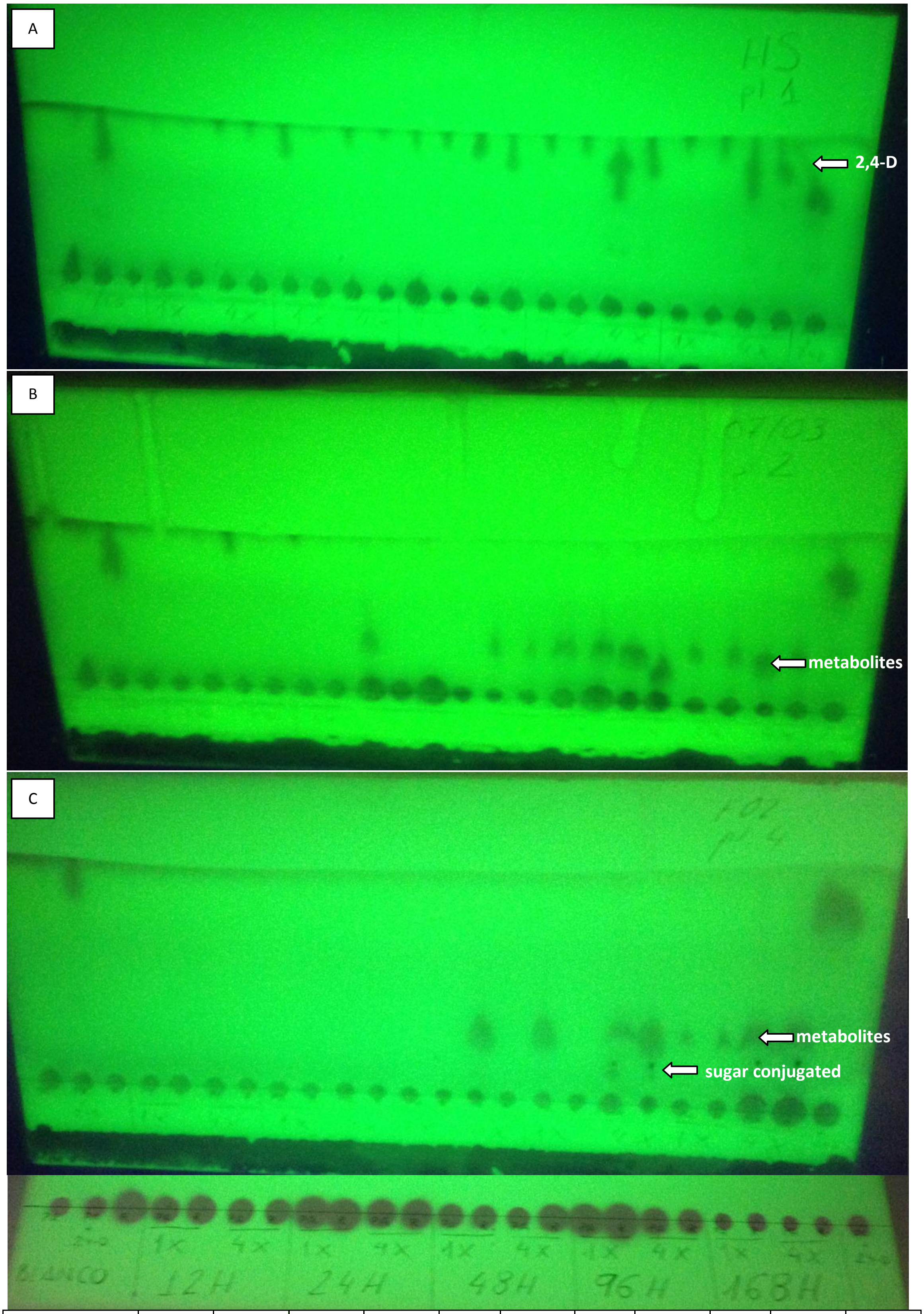

0 hat check	1x	4x	1x	4x	1x	4x	1x	4x	1x	4x	2,4-D
	12 HAT		24 HAT		48 HAT		96 HAT		168 HAT		

FIGURE 1. Metabolism of 2,4-D in *Papaver rhoeas* plants. Extracts from 2,4-D-treated plants at two doses (1x, 600 g a.i./ha; 4x 2400 g a.i./ha) were analysed by TLC, alongside a 2,4-D standard (dot on bottom at the most right-hand side of each plate). Representative TLC separation of extracts from the susceptible (A), only 2,4-D resistant (B) and multiple resistant (C) populations, harvested from 0 h (first three dots on the most left-hand side), 12, 24, 48, 96 and 168 h after application of 2,4-D to leaves of intact plants. Representative TLC plates from three independent experiments are shown.
